# Association of Perceived Job Insecurity With Subsequent Memory Function and Decline Among Adults 55 Years or Older in England and the US, 2006 to 2016

**DOI:** 10.1001/jamanetworkopen.2022.7060

**Published:** 2022-04-13

**Authors:** Xuexin Yu, Kenneth M. Langa, Tsai-Chin Cho, Lindsay C. Kobayashi

**Affiliations:** 1 Center for Social Epidemiology and Population Health, Department of Epidemiology, University of Michigan School of Public Health, Ann Arbor; 2 Department of Internal Medicine, School of Medicine, University of Michigan, Ann Arbor; 3 Institute for Healthcare Policy and Innovation, University of Michigan, Ann Arbor; 4 Veterans Affairs Ann Arbor Center for Clinical Management Research, Ann Arbor, Michigan; 5 Survey Research Center, Institute for Social Research, University of Michigan, Ann Arbor; 6 MRC (Medical Research Council)/Wits Rural Public Health and Health Transitions Research Unit (Agincourt), School of Public Health, Faculty of Health Sciences, University of the Witwatersrand, Johannesburg, South Africa

## Abstract

**Importance:**

Intensified global economic competition and recent financial crises, including those associated with the COVID-19 pandemic, have contributed to uncertainty about job security. However, little is known about the association of perceived job insecurity with memory function and decline among older adults.

**Objectives:**

To investigate the association between perceived job insecurity and subsequent memory function and rate of memory decline among older adults in the US and England.

**Design, Setting, and Participants:**

This 10-year prospective population-based cohort study used data from the US Health and Retirement Study (HRS) and the English Longitudinal Study of Ageing (ELSA) collected from 2006 to 2016. Participants included 9538 adults 55 years or older. Data were analyzed from August 1 to 31, 2021.

**Exposures:**

Perceived job insecurity (yes vs no) at baseline.

**Main Outcomes and Measures:**

Episodic memory *z* scores at baseline and rate of decline during the follow-up.

**Results:**

Among the 9538 study participants, the mean (SD) age at baseline was 60.97 (6.06) years, and 4981 (52.22%) were women. A total of 2320 participants (24.32%) reported job insecurity at baseline (1088 of 3949 [27.55%] in England and 1232 of 5589 [22.04%] in the US). Perceived job insecurity after 55 years of age was associated with lower baseline memory *z* scores in the fully adjusted model (β = −0.04 [95% CI, −0.08 to −0.01]) but not with rate of memory decline (β = 0.01 [95% CI, −0.01 to 0.01]). The association appeared to be stronger in the US than in England (job insecurity × US, β = −0.05 [95% CI, −0.11 to 0.02]), but the estimate was imprecise, potentially owing to low statistical power.

**Conclusions and Relevance:**

The findings of this cohort study suggest that exposure to job insecurity in middle to late life was associated with worse memory function among older adults in the US and England. This association may vary across socioeconomic and social welfare contexts, although future studies with large samples from diverse socioeconomic settings are warranted.

## Key Points

**Question:**

Is perceived job insecurity associated with memory function and rate of decline in England and the US?

**Findings:**

In this cohort study of 9538 participants, exposure to perceived job insecurity in middle to late life was associated with lower memory scores. This association appeared to be stronger in the US than in England, although the estimate was imprecise.

**Meaning:**

These findings suggest that exposure to job insecurity in middle to late life may be a salient risk factor for memory aging, and this association may vary across countries with diverse social welfare safety nets.

## Teaser

This cohort study investigates the association between perceived job insecurity and subsequent memory aging among adults 55 years or older and compares findings in the US vs England to investigate whether country-specific contextual factors modify the observed association.

## Introduction

Intensified global economic competition and recent financial crises, including those associated with the COVID-19 pandemic, have contributed to uncertainty about job security.^1,2,3,4^ Unlike periods of unemployment,^4,5,6^ perceived job insecurity may not be a socially visible event and has been recognized as a salient chronic psychological stressor because individuals may have limited coping strategies for the experience of uncertainty about whether or not the feared layoffs will actually occur.^7,8,9^

Perceived job insecurity has been associated with multiple adverse health outcomes such as increased blood pressure,^10^ weight changes,^10,11^ worse self-rated health,^8,10,12^ psychiatric disorders,^13^ stroke,^10^ and cardiovascular disease.^14,15^ The health effects of perceived job insecurity may accumulate over time, resulting in more permanent adverse health consequences.^7,8,11^ Although perceived job insecurity has been associated with hypertension, stroke, and cardiovascular diseases,^1,7,11,12,14^ which are implicated in the etiology of vascular dementias and Alzheimer disease,^16^ little is known about its association with memory aging among older adults.

Emerging cross-national studies on the health effects of perceived job insecurity across diverse social welfare contexts^14,17^ have yielded inconsistent findings. Cross-country heterogeneity exists in socioeconomic contextual factors such as reemployment opportunities, unemployment benefits, and access to other governmental public safety nets, which are thought to interact with residents’ fears of potential job insecurity.^14^ Although the US shares similar cultures, languages, and economic systems with England, the US provides limited social welfare systems and has greater income inequality than England.^18,19^ For example, England provides universal health coverage and a more generous income maintenance system than the US, which may protect its residents from the adverse health effects of job losses.^19,20^ However, it is unclear whether the association between perceived job insecurity and cognitive aging among older adults in the labor force differs across social welfare regimes.

First, we aimed to investigate the association between perceived job insecurity and subsequent memory aging among adults 55 years or older in a pooled analysis of data from the US and England. Second, we aimed to compare findings in the US vs England to investigate whether country-specific contextual factors could modify the observed association. We hypothesized that (1) exposure to perceived job insecurity would be associated with a lower level of memory function and a faster rate of memory decline over time, and (2) the association would be stronger in the US than in England, because England provides stronger government social safety nets than the US.^20^

## Methods

### Data Source and Study Design

Data for this cohort study were from the US Health and Retirement Study (HRS) and the English Longitudinal Study of Ageing (ELSA) from 2006 to 2016. The US HRS is a national biennial longitudinal study of more than 20 000 individuals older than 50 years since 1992.^21^ The ELSA is a nationally representative longitudinal household survey of approximately 15 000 individuals older than 50 years since 2002.^22^ The ELSA and HRS use comparable survey methods and questionnaires,^20^ the details of which have been documented elsewhere.^21,22^ The HRS was approved by the University of Michigan Institutional Review Board, and the ELSA was approved by the National Health Service National Research and Ethics Service in England. Written informed consent was obtained from all study participants. This study followed the Strengthening the Reporting of Observational Studies in Epidemiology (STROBE) reporting guideline.

**Figure 1.**
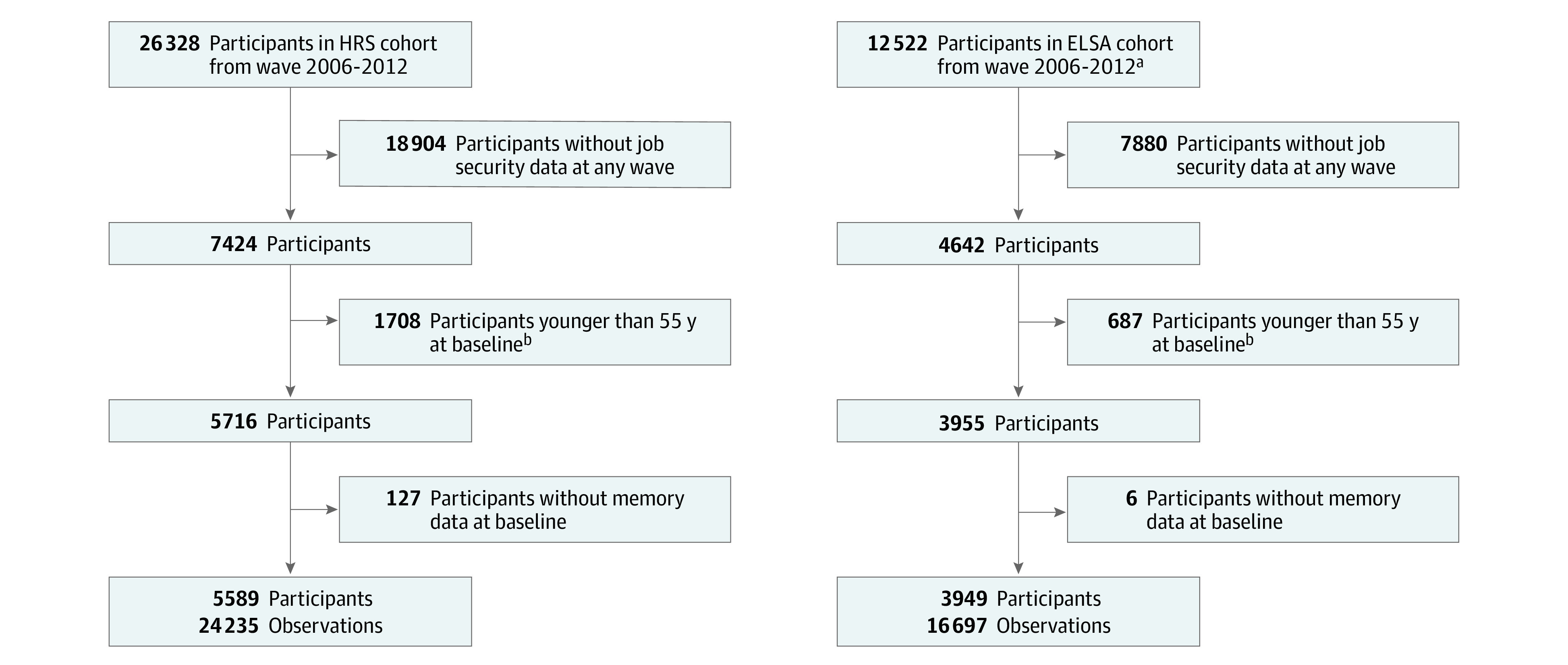
Study Flow Diagram Adults 55 years or older from the English Longitudinal Study of Ageing (ELSA) and the US Health and Retirement Study (HRS) were included. ^a^Indicates core members living in private household. We excluded partners of core members and those living in institutions because of missing survey weights in ELSA. ^b^We identified the earliest wave from 2006 to 2012, when individuals 55 years or older provided job insecurity data.

We used a dynamic longitudinal study design with perceived job insecurity (yes vs no) measured at each participant’s baseline, and episodic memory *z* scores (immediate and delayed recall) assessed at baseline and subsequent years of biennial follow-up in each cohort. Individuals 55 years or older at baseline with data on job insecurity, memory scores, and sampling weights were eligible for inclusion (Figure 1). We chose 55 years of age for eligibility because it is the youngest age to be nationally representative for all included years in the HRS and ELSA. A proportion of individuals in the HRS and ELSA were randomly selected to participate in the self-completion modules, which contained question items on perceived job insecurity from 2006 to 2012. Because the timing of the self-completion module administrations varied, we used a dynamic longitudinal study design and defined the baseline as the earliest wave (2006, 2008, 2010, and 2012) when individuals 55 years or older provided data on job insecurity (eFigure in the Supplement). This study included 9538 individuals (5589 in the HRS and 3949 in the ELSA), contributing 40 932 observations from 2006 to 2016 (24 235 in the HRS and 16 697 in the ELSA).

### Measures

#### Exposure

Exposure to perceived job insecurity was measured at baseline. All individuals selected for the self-completion modules who were currently working were asked to agree or disagree with the statement: “My job security is poor.” The available response options included strongly disagree, disagree, agree, and strongly agree. We identified individuals with perceived job insecurity (yes or no) as those who agreed or strongly agreed with this statement. We combined those who agreed and strongly agreed because a limited number of individuals strongly agreed (666 [6.98%]). In addition, we measured individuals’ residence (US vs England) to reflect country-specific social context.

#### Outcome

Episodic memory scores (0-20 points) were assessed at baseline and biennially in subsequent years of follow-up by summing immediate word recall scores (0-10 points) and delayed word recall scores (0-10 points). Immediate word recall required the participants to immediately recall a list of 10 nouns provided by the interviewers in any order.^23^ For delayed word recall, the participants were asked to recall the 10 nouns after a 5-minute delay.^23^ We calculated memory *z* scores using the mean (SD) of the overall episodic memory scores at the baseline wave from the pooled analytic data set.^24^

#### Covariates

We included sociodemographic characteristics, lifestyle attributes, comorbid disease history, and depression, all measured at baseline, as potential confounders between job insecurity and memory function.^11,12,13,16,20^ Socioeconomic characteristics included age, sex, marital status (partnered, separated or divorced, widowed, or never married), foreign-born status (outside the US for HRS; outside the UK for ELSA), self-identified race and ethnicity (White vs other [in the HRS, including American Indian or Alaska Native, Asian or Pacific Islander, Black or African American, combination of Black and American Indian, Hispanic or Latino, and others; in ELSA, including Asian, Asian British citizens, Black, Black British citizens, mixed ethnic group (Asian, Black, White, or any other), and any other]. We combined these racial and ethnic groups because of the heterogeneity in the US and England), educational level (less than upper secondary, upper secondary and vocational training, or tertiary, according to the International Standard Classification of Education),^20^ current occupation (lower-skilled or higher-skilled employment, according to the International Standard Classification of Occupation [details in eTables 1 and 2 in the Supplement]),^25^ and wealth in quintiles within each country.^20^ Lifestyle attributes included alcohol consumption (ever or never), tobacco use (ever or never), and body mass index (calculated as weight in kilograms divided by height in meters squared; categories of <18.5, 18.5-24.9, 25.0-29.9, or >29.9). Comorbid diseases were self-reported, including hypertension (yes or no), diabetes (yes or no), stroke (yes or no), cardiovascular disease (yes or no), and cancer (yes or no). Potential depressive symptoms (yes or no) were identified according to scores of 3 or greater in the 8-item Center for Epidemiologic Studies Depression Scale.^20^ Last, we included baseline year (2006, 2008, 2010, or 2012) as a covariate to identify unmeasured baseline period effects, such as those associated with the Great Recession.^2,26,27,28^

### Statistical Analysis

Data were analyzed from August 1 to 31, 2021. We compared baseline characteristics according to perceived job insecurity overall and across the HRS and ELSA study populations (upaired *t* test, Pearson χ^2^ test, and Wilcoxon rank-sum test). Next, we ran multivariable-adjusted linear mixed-effects regression models with person-specific random intercepts and slopes to investigate the associations between perceived job insecurity and initial memory scores at baseline and subsequent rate of memory decline over time.^29^ We included an interaction term between job insecurity status and years of follow-up (ranging from 0 to 10) to investigate whether job insecurity was associated with memory decline. In addition, we repeated the models including a statistical interaction term between job insecurity and country of residence (US vs England) to investigate whether the association between job insecurity and memory varied by country. We tested a 3-way interaction among job insecurity, country of residence (US vs England), and years of follow-up, which was not statistically significant (*P* > .05) and therefore removed from the main analyses. We also included a squared term for years of follow-up to examine whether there was a nonlinear rate of decline over time in the memory *z* scores.

We conducted 3 sets of sequential models. Model 1 was adjusted for baseline year, age, sex, marital status, foreign-born status, and race and ethnicity (demographic adjustment). Model 2 was additionally adjusted for educational levels, current occupation, and household wealth (sociodemographic adjustment). Model 3 was additionally adjusted for lifestyle attributes, comorbid disease history, and depression (fully adjusted). Multiple imputation by chained equations was performed in the pooled data set to impute values of missing covariates. We produced 5 imputed data sets conditional on all other observed variables in the analytic model.^30,31,32^

We performed several sensitivity analyses. First, baseline survey weights were applied to account for the complex survey designs of the HRS and ELSA. Second, we repeated the analyses with inverse probability of censoring weights to minimize potential bias from selective attrition during follow-up. Third, we restricted the study sample to those aged 55 to 64 years and repeated the modeling analyses because individuals 65 years or older in the US may have more social welfare resources than those younger than 65 years.^20^ In addition, we restricted the study sample to individuals with at least upper secondary and vocational training and those with no history of hypertension, diabetes, stroke, cardiovascular disease, cancer, and depressive symptoms at baseline to rule out potential reverse causality, because individuals with lower educational attainment and chronic comorbidities may have lower cognitive function before baseline and may be more likely to perceive job insecurity.^33,34,35^ Moreover, we repeated modeling analyses restricted to those who did not experience job dismissal before the exposure period to rule out unmeasured confounding by objective job insecurity. Finally, we compared baseline characteristics between included and excluded participants. All analyses were performed with Stata/SE, version 15.0 (StataCorp LLC). Two-sided *P* < .05 indicated statistical significance.

## Results

Among the 9538 study participants, the mean (SD) age at baseline was 60.97 (6.06) years; 4981 (52.22%) were women and 4557 (47.78%) were men. Table 1 presents the baseline characteristics of the sample by perceived job insecurity overall and across countries. A total of 2320 participants (24.32%) reported job insecurity at baseline (1088 of 3949 [27.55%] in England and 1232 of 5589 [22.04%] in the US). Participants with job insecurity were more likely than those who did not report job insecurity to have poor memory function (mean [SD] score, 10.93 [3.12] vs 11.27 [3.03]; *P* < .001), higher body mass index (942 of 2320 [40.60%] vs 2657 of 7218 [36.81%]; *P* < .001), more depressive symptoms (491 of 2320 [21.16%] vs 864 of 7218 [11.97%]; *P* < .001), and a history of smoking (1365 of 2320 [58.84%] vs 4010 of 7218 [55.55%]; *P* = .02), diabetes (302 of 2320 [13.02%] vs 785 of 7218 [10.87%]; *P* < .001), and cardiovascular disease (304 of 2320 [13.10%] vs 835 of 7218 [11.57%]; *P* = .04) (Table 1). They were also less likely to be women (1161 of 2320 [50.04%] vs 3820 of 7218 [52.92%]; *P* = .02), older (mean [SD] age, 60.39 [5.76] vs 61.16 [6.14] years; *P* < .001), and White (1933 of 2320 [83.32%] vs 6263 of 7218 [86.77%]; *P* < .001) and to have a higher-skilled occupation (745 of 2320 [32.11%] vs 2886 of 7218 [39.98%]; *P* < .001) than those without job insecurity.

**Table 1.** Baseline Characteristics of Study Sample According to Job Insecurity in the US (HRS) and England (ELSA), 2006 to 2016^a^

Characteristic	Pooled cohort				HRS cohort					ELSA cohort			
	Total (N = 9538)	Job insecurity		*P* value	Total (n = 5589)	Job insecurity			*P* value	Total (n = 3949)	Job insecurity		*P* value
		No (n = 7218)	Yes (n = 2320)			No (n = 4357)		Yes (n = 1232)			No (n = 2861)	Yes (n = 1088)	
Memory scores, mean (SD)	11.19 (3.06)	11.27 (3.03)	10.93 (3.12)	<.001^b^	10.85 (2.98)		10.95 (2.95)	10.49 (3.07)	<.01^b^	11.66 (3.10)	11.75 (3.10)	11.43 (3.11)	<.001^b^
Age, y													
Mean (SD)	60.97 (6.06)	61.16 (6.14)	60.39 (5.76)	<.001^b^	62.27 (6.63)		62.49 (6.69)	61.46 (6.33)	<.001^b^	59.14 (4.56)	59.13 (4.49)	59.17 (4.75)	<.001^b^
Median (range)	59 (55-97)	59 (55-97)	58 (55-94)		60 (55-97)		61 (55-97)	59 (55-94)		58 (55-86)	58 (55-86)	58 (55-84)	
Sex													
Women	4981 (52.22)	3820 (52.92)	1161 (50.04)	.02^c^	2994 (53.57)		2344 (53.80)	650 (52.76)	.52^c^	1987 (50.32)	1476 (51.59)	511 (46.97)	<.001^c^
Men	4557 (47.78)	3398 (47.08)	1159 (49.96)		2595 (60.96)		2013 (46.20)	582 (47.24)		1962 (49.68)	1385 (49.41)	577 (53.03)	
Race and ethnicity													
White	8196 (85.93)	6263 (86.77)	1933 (83.32)	<.001^c^	4367 (78.14)		3474 (79.73)	893 (72.48)	<.001^c^	3829 (96.96)	2789 (97.48)	1040 (95.59)	<.001^c^
Other^d^	1342 (14.07)	955 (13.23)	387 (16.68)		1222 (21.86)		883 (20.27)	349 (28.33)		120 (3.04)	72 (2.52)	48 (4.41)	
Foreign-born status	383 (4.01)	268 (3.71)	115 (4.96)	<.001^c^	23 (0.41)		19 (0.44)	4 (0.32)	.59^c^	360 (9.12)	249 (8.70)	111 (10.20)	.14^c^
Marital status													
Partnered	7157 (75.04)	5497 (76.16)	1660 (71.55)	<.001^c^	3991 (71.41)		3172 (72.80)	819 (66.48)	<.001^c^	3166 (80.17)	2325 (81.27)	841 (77.30)	<.001^c^
Separated or divorced	1316 (13.80)	943 (13.06)	373 (16.08)		880 (15.75)		644 (14.78)	236 (19.15)		436 (11.04)	299 (10.45)	137 (12.59)	
Widowed	636 (6.67)	485 (6.72)	151 (6.51)		478 (8.55)		368 (8.45)	110 (8.93)		158 (4.00)	117 (4.09)	41 (3.77)	
Never married	429 (4.50)	293 (4.06)	136 (5.86)		240 (4.29)		173 (3.97)	67 (5.44)		189 (4.79)	120 (4.19)	69 (6.34)	
Educational level													
Less than upper secondary	1345 (14.10)	951 (13.18)	394 (16.98)	<.001^e^	601 (10.75)		414 (9.50)	187 (15.18)	<.001^e^	744 (18.84)	537 (18.77)	207 (19.03)	.23^e^
Upper secondary and vocational training	5007 (52.50)	3798 (52.62)	1209 (52.11)		3276 (58.62)		2547 (58.46)	729 (59.17)		1731 (43.83)	1251 (43.73)	480 (44.12)	
Tertiary	2442 (25.60)	1943 (26.92)	499 (21.51)		1712 (30.63)		1396 (32.04)	316 (25.65)		730 (18.49)	547 (19.12)	183 (16.82)	
Household wealth, quintile													
First (poorest)	1596 (16.73)	1106 (15.32)	490 (21.12)	<.001^e^	872 (15.60)		603 (13.84)	269 (21.83)	<.001^e^	724 (18.33)	503 (17.58)		.02^e^
Second	1798 (18.85)	1317 (18.25)	481 (20.73)		1211 (21.67)		902 (20.70)	309 (25.08)		587 (14.86)	415 (14.51)	172 (15.81)	
Third	2022 (21.20)	1519 (21.04)	503 (21.68)		1198 (21.43)		930 (21.34)	268 (21.75)		824 (20.87)	589 (20.59)	235 (21.60)	
Fourth	2045 (21.44)	1597 (22.13)	448 (19.31)		1159 (20.74)		955 (21.92)	204 (16.56)		886 (22.44)	642 (22.44)	244 (22.43)	
Fifth (richest)	2031 (21.29)	1644 (22.78)	387 (16.68)		1149 (20.56)		967 (22.19)	182 (14.77)		882 (22.33)	677 (23.66)	205 (18.84)	
Higher-skilled occupation (vs lower-skilled)	3631 (38.07)	2886 (39.98)	745 (32.11)	<.001^c^	2088 (37.36)		1721 (39.50)	367 (29.79)	<.001^c^	1543 (39.07)	1165 (40.72)	378 (34.74)	<.001^c^
Smoking history (vs never)	5375 (56.35)	4010 (55.55)	1365 (58.84)	.02^c^	3054 (54.64)		2346 (53.84)	708 (57.470	.04^c^	2321 (58.77)	1664 (58.16)	657 (60.39)	.19^c^
Alcohol consumption (vs none)	7159 (75.06)	5426 (75.17)	1733 (74.70)	.64^c^	3503 (62.68)		2760 (63.35)	743 (60.31)	.05^c^	3656 (92.58)	2666 (93.18)	990 (90.99)	.02^c^
BMI													
<18.5	70 (0.73)	52 (0.72)	18 (0.77)	<.001^e^	42 (0.75)		33 (0.76)	9 (0.73)	<.001^e^	28 (0.71)	19 (0.66)	9 (0.83)	.21^e^
18.5-24.9	2269 (23.79)	1752 (24.27)	517 (22.28)		1386 (24.80)		1130 (25.93)	256 (20.78)		883 (22.36)	622 (21.74)	261 (23.99)	
25.0-29.9	3576 (37.49)	2743 (38.00)	833 (35.91)		2117 (37.88)		1666 (38.24)	451 (36.61)		1459 (36.95)	1077 (37.64)	382 (35.11)	
>29.9	3599 (37.73)	2657 (36.81)	942 (40.60)		2041 (36.52)		1526 (35.02)	515 (41.80)		1558 (39.45)	1131 (39.53)	427 (39.25)	
Hypertension (vs no)	3859 (40.46)	2884 (39.95)	975 (42.03)	.07^c^	2641 (47.25)		2020 (46.36)	621 (50.41)	.01^c^	1218 (30.84)	864 (30.20)	354 (32.54)	.16^c^
Diabetes (vs no)	1087 (11.40)	785 (10.87)	302 (13.02)	<.001^c^	883 (15.80)		651 (14.94)	232 (18.83)	<.001^c^	204 (5.17)	134 (4.68)	70 (6.43)	.03^c^
Stroke (vs no)	230 (2.41)	168 (2.33)	62 (2.67)	.35^c^	185 (3.31)		134 (3.08)	51 (4.14)	.06^c^	45 (1.14)	34 (1.19)	11 (1.01)	.64^c^
Cardiovascular disease (vs no)	1139 (11.94)	835 (11.57)	304 (13.10)	.04^c^	759 (13.58)		561 (12.87)	198 (16.07)	<.001^c^	380 (9.62)	274 (9.58)	106 (9.74)	.86^c^
Cancer (vs no)	734 (7.70)	561 (7.77)	173 (7.46)	.62^c^	527 (9.43)		414 (9.50)	113 (9.17)	.73^c^	207 (5.24)	147 (5.14)	60 (5.51)	.64^c^
Depressive symptoms (vs no)	1355 (14.21)	864 (11.97)	491 (21.16)	<.001^c^	798 (14.28)		509 (11.68)	289 (23.46)	<.001^c^	557 (14.10)	355 (12.41)	202 (18.57)	<.001^c^

Abbreviations: BMI, body mass index (calculated as weight in kilograms divided by height in meters squared); ELSA, English Longitudinal Study of Ageing; HRS, US Health and Retirement Study.

^a^ Unless otherwise indicated, data are expressed as number (%) of participants. Owing to missing data, numbers may total less than column headings. Data were missing for educational level (744 [7.80%]), household wealth (46 [0.48%]), smoking history (39 [0.41%]), and BMI (24 [0.25%]). Percentages have been rounded and may not total 100.

^b^ Calculated using the unpaired *t* test.

^c^ Calculated using the Pearson χ^2^ test.

^d^ Other racial and ethnic groups in the HRS included American Indian or Alaska Native, Asian or Pacific Islander, Black or African American, combination of Black and American Indian, Hispanic or Latino, and others; other racial and ethnic groups in the ELSA included Asian, Asian British citizens, Black, Black British citizens, mixed ethnic group (Asian, Black, White, or any other), and any other group.

^e^ Calculated using Wilcoxon rank-sum test.

**Figure 2.**
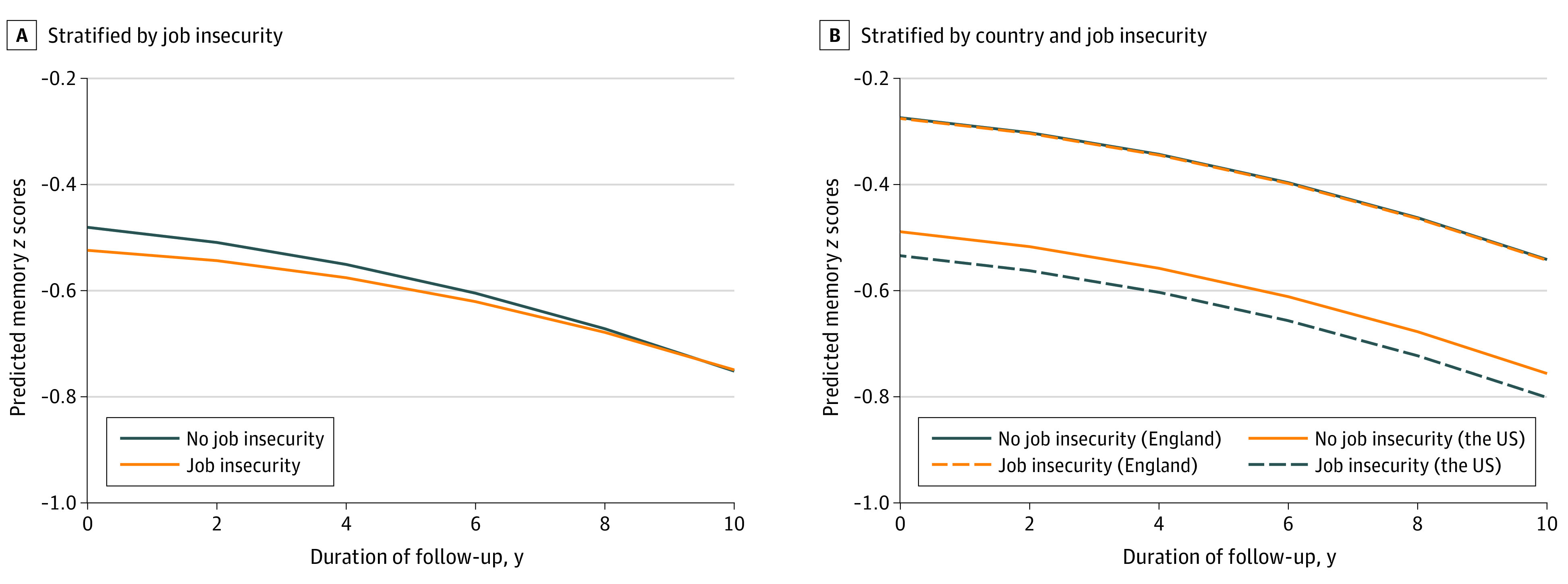
Estimated Memory *z* Scores by Job Insecurity and Country Includes adults 55 years or older from the English Longitudinal Study of Ageing and the US Health and Retirement Study, 2006 to 2016. Memory *z* scores are estimated in model 3 in Table 2. Covariates in model 3 were set to the following values: 60 years of age, female sex, partnered, White race and ethnicity, foreign-born, less than upper secondary educational level, first quintile of household wealth, higher-skilled occupation, smoked, alcohol consumption, body mass index of 18.5 to 24.9 (calculated as weight in kilograms divided by height in meters squared), and diagnosed with hypertension, diabetes, stroke, cancer, cardiovascular disease, and depressive symptoms.

Table 2 and Figure 2 present results from pooled analyses incorporating data from both the US and England. Perceived job insecurity after 55 years of age was associated with lower memory *z* scores at baseline in model 1 (β = −0.11 [95% CI, −0.15 to −0.07]). These associations were attenuated but remained statistically significant in the fully adjusted model 3 (β = −0.04 [95% CI, −0.08 to −0.01]). However, perceived job insecurity was not associated with rate of memory decline (models 1-3, β = 0.01 [95% CI, −0.01 to 0.01]). The estimate from the squared term for years of follow-up indicated nonlinear memory decline during follow-up (model 3, β = −0.002 [95% CI, −0.01 to −0.01]).

**Table 2.** Multivariable-Adjusted Mixed-Effects Linear Regression Analyses of the Association Between Job Insecurity and Memory Function and Decline From 2006 to 2016 Using Imputed Data Sets^a^

Variable	Model 1		Model 2		Model 3	
	β (95% CI)	*P* value	β (95% CI)	*P* value	β (95% CI)	*P* value
**Pooled analyses with interaction between job insecurity and years of follow-up**						
Job insecurity (yes vs no)	−0.11 (−0.15 to −0.07)	<.001	−0.06 (−0.10 to −0.02)	<.001	−0.04 (−0.08 to −0.01)	.04
Years of follow-up	−0.01 (−0.02 to −0.01)	.01	−0.01 (−0.02 to −0.01)	.01	−0.01 (−0.02 to −0.01)	.01
Job insecurity × years of follow-up	0.01 (−0.01 to 0.01)	.11	0.01 (−0.01 to 0.01)	.13	0.01 (−0.01 to 0.01)	.13
Years of follow-up^2^	−0.01 (−0.01 to −0.01)	<.001	−0.01 (−0.01 to −0.01)	<.001	−0.002 (−0.01 to −0.01)	<.001
**Pooled analyses with interaction between job insecurity and country of residence**						
Job insecurity (yes vs no)	−0.04 (−0.10 to 0.01)	.13	−0.01 (−0.06 to 0.04)	.66	−0.01 (−0.05 to 0.05)	.99
US (vs England)	−0.11 (−0.15 to −0.07)	<.001	−0.21 (−0.25 to −0.18)	<.001	−0.17 (−0.21 to −0.13)	<.001
Job insecurity × US	−0.09 (−0.17 to −0.02)	.01	−0.06 (−0.13 to 0.01)	.11	−0.05 (−0.11 to 0.02)	.19
Years of follow-up	−0.01 (−0.02 to −0.01)	.02	−0.01 (−0.02 to −0.01)	.01	−0.01 (−0.01 to −0.01)	.01
Years of follow-up^2^	−0.01 (−0.01 to −0.01)	<.001	−0.01 (−0.01 to −0.01)	<.001	−0.01 (−0.01 to −0.01)	<.001
Job insecurity × US × years of follow-up^b^	NA	.16	NA	.14	NA	.14

Abbreviation: NA, not applicable.

^a^ Data are from the English Longitudinal Study of Ageing and US Health and Retirement Study (N = 9538). Multiple imputation by chained equations was performed to impute missing values of educational level, household wealth, occupation, smoking history, and body mass index. Memory scores were *z* score standardized according to the mean and SD at baseline. Model 1 adjusted for baseline year, baseline age, sex, marital status, race and ethnicity, and foreign-born status; model 2, for baseline year, baseline age, sex, marital status, race and ethnicity, foreign-born status, occupation, household wealth, and educational level; and model 3, for baseline year, baseline age, sex, marital status, race and ethnicity, foreign-born status, occupation, household wealth, educational level, alcohol consumption, smoking history, body mass index, hypertension, diabetes, stroke, cardiovascular diseases, cancer, and depressive symptoms.

^b^ The 3-way interaction term was not statistically significant in all 3 models and therefore was not included in the main analyses.

When we tested an interaction term between job insecurity and country of residence, the association between perceived job insecurity and memory function appeared to be stronger in the US than in England (model 1, β = −0.09 [95% CI, −0.17 to −0.02]). However, approximately 30% of this cross-national difference was explained by heterogeneity in socioeconomic characteristics, including wealth, educational level, and occupation (model 2, β = −0.06 [95% CI, −0.13 to 0.01]; model 3, β = −0.05 [95% CI, −0.11 to 0.02]) (Table 2).

The estimates from the sensitivity analyses with additional sampling weights and inverse probability weights were similar to those from the main analyses (eTables 3 and 4 in the Supplement). Sensitivity analyses restricted to individuals aged 55 to 64 years, individuals with higher educational attainment, and those with no history of hypertension, diabetes, stroke, cancer, cardiovascular disease, and depressive symptoms were consistent with those observed in the main analyses, although the 95% CIs were wide, potentially owing to the increased variance imposed by weighting and sample restriction (eTables 5-7 in the Supplement). Analyses restricted to individuals who did not experience dismissal from a job before the baseline provided results similar to those in main analyses (eTable 8 in the Supplement). Baseline characteristics of the included and excluded participants are provided in eTable 9 in the Supplement.

## Discussion

In this pooled longitudinal cohort study in the US and England, job insecurity after 55 years of age was associated with lower memory function but not with the rate of memory decline over time. The negative association between job insecurity and memory function was stronger in the US than in England, although the estimates were somewhat imprecise. Our findings suggest that exposure to perceived job insecurity could be an important factor associated with memory aging.

### Comparison With Existing Evidence

Although the biological plausibility for the association under study is strong,^3,7,8,9,11,12,14,17,36^ limited empirical evidence is available for the association of perceived job insecurity and memory aging. Exposure to job insecurity, especially if accumulated over time, may impose psychological stress, leading to worse sleep quality, anxiety, and depressive symptoms.^8,36^ Existing studies have also demonstrated the relation of perceived job insecurity with hypertension, obesity, and cardiovascular diseases.^10,14,15,37^ All these conditions have been implicated in the etiology of Alzheimer disease and related dementias.^16,38^

Although the associations with job insecurity appear to be strong for nonfatal myocardial infarction (risk ratio, 1.89),^15^ depressive symptoms (risk ratio, 2.04),^39^ and hypertension (odds ratio, 1.60),^37^ we observed a modest association between perceived job insecurity and memory function. The magnitude of this association was similar to the mean 1-year memory aging observed during the 10-year follow-up period in this sample (−0.04 SD units for job insecurity vs −0.01 SD units for 1 year of follow-up and −0.002 SD units for a squared term for years of follow-up, as shown in model 3) (Table 2). However, our findings were in line with those of a prior meta-analysis^14^ that incorporated 13 cohort studies with more than 17 million individuals, demonstrating a risk ratio of 1.19 (95% CI, 1.00-1.42) for coronary heart disease among those perceiving job insecurity compared with those without job insecurity. These findings suggest that perceived job insecurity may be a chronic stressor that has modest effects on middle-to-later life memory function.

The observed association between perceived job insecurity and memory function appeared to be modified by country-specific social contexts, which are consistent with existing studies indicating that health disparities in the US are more profound than those in England and other high-income countries.^19,20,40,41,42^ Although differences in social welfare regimes are a possible explanation for the observed effect size modification by country, population differences in psychosocial factors such as stress may also play a role. Existing evidence has also indicated that compared with those in England, individuals residing in the US have higher-level C-reactive protein and fibrinogen, which are thought to connect closely with psychosocial stress.^43^ The more generous income maintenance system and comprehensive access to health care in England may help reduce psychosocial stress associated with fear of layoffs and thus diminish the negative health outcomes associated with perceived job insecurity.^19^ However, our estimations were imprecise with the 95% CIs that crossed the null, potentially owing to the modest magnitude of association and limited statistical power. Further research with a larger sample size is warranted.

### Strengths and Limitations

To the best of our knowledge, this study is one of the first to investigate the association between job insecurity in later life and memory function during aging. We measured job insecurity after 55 years of age, a key period in the life course when the potential of getting a new job is reduced in the years leading up to retirement.^44,45,46^ Exposure to job insecurity at this period may impose greater psychological and financial stress than in earlier life periods^4,5,47,48^ and thus may be a salient risk factor for cognitive health during aging.

This study has several limitations. The differences in measurement error in all variables used may exist between the 2 cohorts, which we are unable to minimize, although we harmonized data from the 2 cohorts to the best of our ability and in line with prior research.^20^ Our study may underestimate cross-national differences in the association between perceived job insecurity and memory function, because individuals may have systematically different thresholds for reporting perceived job insecurity across cultures and social welfare regimes. Individuals from England might be more conservative in reporting perceived job insecurity given the stronger social safety net, although in our study, the prevalence of job insecurity was slightly higher in England (27.55%) than in the US (22.04%), potentially because of the exclusion of partners with missing sampling weights in the ELSA. Because we required participants to have data on perceived job insecurity (an interview question for current employees only), participants who were unemployed during the exposure period were not eligible for inclusion. These individuals may have experienced extreme levels of job insecurity before an unemployment episode, which would not have been reported; therefore, this study may underestimate the association between exposure to job insecurity and memory. Furthermore, although we included multiple lifestyle behaviors and comorbid disease history measured at baseline as potential confounders of the observed association, these characteristics could be affected by lifelong cumulative exposure to job insecurity before baseline, leading to overadjustment in fully adjusted model 3 (Table 2). In addition, although exposure to job insecurity may accumulate or change over time,^7,8,11^ we were unable to investigate the association of cumulative job insecurity exposure with memory outcomes because of data limitations. Further research with repeated measures of perceived job insecurity is warranted. Moreover, our findings may be subject to reverse causation, although we conducted sensitivity analyses restricted to healthy individuals and those with higher educational attainment to rule out this potential as best possible. Last, although we pooled data from the HRS and ELSA, we were unable to provide precise estimates of the association between job insecurity and memory function, because they appear to be modest in magnitude and may require a larger sample size to detect.^14^

## Conclusions

The findings of this cohort study suggest that exposure to perceived job insecurity may be associated with poorer cognitive health during aging, and the magnitude of this association may vary across countries and social welfare regimes. Future studies with larger sample sizes from diverse socioeconomic settings are warranted to better understand this complex relationship.

## Notes

Supplement. **eMethods.** Creation of Inverse Probability of Censoring Weights (IPCWs) **eFigure.** Study Design and Distribution of Study Sample From 2006-2016 **eTable 1.** Classification and Definition of Occupations in HRS **eTable 2.** Classification and Definition of Occupations in ELSA **eTable 3.** Sampling-Weighted Linear Mixed-Effects Regression Analyses of the Association Between Job Insecurity and Memory and Decline From 2006-2016, US (HRS) and England (ELSA) (N = 9538) **eTable 4.** Attrition-Weighted Linear Mixed-Effects Regression Analyses of the Association Between Job Insecurity and Memory and Decline From 2006-2016, the US (HRS) and England (ELSA) (N = 9538) **eTable 5.** Restricted Multivariable-Adjusted Linear Mixed-Effects Regression Analyses of the Association Between Job Insecurity and Memory and Decline Among Individuals Aged 55-64 From 2006-2016, the US (HRS) and England (ELSA) (N = 7295) **eTable 6.** Restricted Multivariable-Adjusted Linear Mixed-Effects Regression Analyses of the Association Between Job Insecurity and Memory and Decline Among Participants With at Least Upper Secondary and Vocational Training From 2006-2016, the US (HRS) and England (ELSA) (N = 7449) **eTable 7.** Restricted Multivariable-Adjusted Linear Mixed-Effects Regression Analyses of the Association Between Job Insecurity and Memory and Decline Among Participants With No History of Diabetes, Hypertension, Stroke, Cardiovascular Disease, Cancer, and Depressive Symptoms at Baseline, the US (HRS) and England (ELSA) (N = 3990) **eTable 8.** Restricted Multivariable-Adjusted Linear Mixed-Effects Regression Analyses of the Association Between Job Insecurity and Memory and Decline Among Participants That Were Not Dismissed Before Baseline, 2006-2016, the US (HRS) and England (ELSA) (N = 8232) **eTable 9.** Baseline Characteristics of the Included Study Sample and Excluded Individuals, the US (HRS) and England (ELSA), 2006-2016
