# Supplementary material for: Association of Perceived Job Insecurity With Subsequent Memory Function and Decline Among Adults 55 Years or Older in England and the US, 2006 to 2016
Source: JAMA Netw Open. 2022 Apr 13;5(4):e227060. doi: 10.1001/jamanetworkopen.2022.7060 (PMC9008497; doi:10.1001/jamanetworkopen.2022.7060)
Supplement: Supplement. — eMethods. Creation of Inverse Probability of Censoring Weights (IPCWs) eFigure. Study Design and Distribution of Study Sample From 2006-2016 eTable 1. Classification and Definition of Occupations in HRS eTable 2. Classification and Definition of Occupations in ELSA eTable 3. Sampling-Weighted Linear Mixed-Effects Regression Analyses of the Association Between Job Insecurity and Memory and Decline From 2006-2016, US (HRS) and England (ELSA) (N = 9538) eTable 4. Attrition-Weighted Linear Mixed-Effects Regression Analyses of the Association Between Job Insecurity and Memory and Decline From 2006-2016, the US (HRS) and England (ELSA) (N = 9538) eTable 5. Restricted Multivariable-Adjusted Linear Mixed-Effects Regression Analyses of the Association Between Job Insecurity and Memory and Decline Among Individuals Aged 55-64 From 2006-2016, the US (HRS) and England (ELSA) (N = 7295) eTable 6. Restricted Multivariable-Adjusted Linear Mixed-Effects Regression Analyses of the Association Between Job Insecurity and Memory and Decline Among Participants With at Least Upper Secondary and Vocational Training From 2006-2016, the US (HRS) and England (ELSA) (N = 7449) eTable 7. Restricted Multivariable-Adjusted Linear Mixed-Effects Regression Analyses of the Association Between Job Insecurity and Memory and Decline Among Participants With No History of Diabetes, Hypertension, Stroke, Cardiovascular Disease, Cancer, and Depressive Symptoms at Baseline, the US (HRS) and England (ELSA) (N = 3990) eTable 8. Restricted Multivariable-Adjusted Linear Mixed-Effects Regression Analyses of the Association Between Job Insecurity and Memory and Decline Among Participants That Were Not Dismissed Before Baseline, 2006-2016, the US (HRS) and England (ELSA) (N = 8232) eTable 9. Baseline Characteristics of the Included Study Sample and Excluded Individuals, the US (HRS) and England (ELSA), 2006-2016 [file jamanetwopen-e227060-s001.pdf]

Supplementary Online Content

Yu X, Langa KM, Cho TC, Kobayashi LC. Association of perceived job insecurity with subsequent memory function and decline among adults 55 years or older in England and the US, 2006 to 2016. *JAMA Netw Open*. 2022;5(4):e227060. doi:10.1001/jamanetworkopen.2022.7060

eMethods. Creation of Inverse Probability of Censoring Weights (IPCWs)
eFigure. Study Design and Distribution of Study Sample From 2006-2016
eTable 1. Classification and Definition of Occupations in HRS
eTable 2. Classification and Definition of Occupations in ELSA
eTable 3. Sampling-Weighted Linear Mixed-Effects Regression Analyses of the Association Between Job Insecurity and Memory and Decline From 2006-2016, US (HRS) and England (ELSA) (N = 9538)
eTable 4. Attrition-Weighted Linear Mixed-Effects Regression Analyses of the Association Between Job Insecurity and Memory and Decline From 2006-2016, the US (HRS) and England (ELSA) (N = 9538)
eTable 5. Restricted Multivariable-Adjusted Linear Mixed-Effects Regression Analyses of the Association Between Job Insecurity and Memory and Decline Among Individuals Aged 55-64 From 2006-2016, the US (HRS) and England (ELSA) (N = 7295)
eTable 6. Restricted Multivariable-Adjusted Linear Mixed-Effects Regression Analyses of the Association Between Job Insecurity and Memory and Decline Among Participants With at Least Upper Secondary and Vocational Training From 2006-2016, the US (HRS) and England (ELSA) (N = 7449)
eTable 7. Restricted Multivariable-Adjusted Linear Mixed-Effects Regression Analyses of the Association Between Job Insecurity and Memory and Decline Among Participants With No History of Diabetes, Hypertension, Stroke, Cardiovascular Disease, Cancer, and Depressive Symptoms at Baseline, the US (HRS) and England (ELSA) (N = 3990)
eTable 8. Restricted Multivariable-Adjusted Linear Mixed-Effects Regression Analyses of the Association Between Job Insecurity and Memory and Decline Among Participants That Were Not Dismissed Before Baseline, 2006-2016, the US (HRS) and England (ELSA) (N = 8232)
eTable 9. Baseline Characteristics of the Included Study Sample and Excluded Individuals, the US (HRS) and England (ELSA), 2006-2016

This supplementary material has been provided by the authors to give readers additional information about their work.

© 2022 Yu X et al. *JAMA Network Open*.

eMethods. Creation of Inverse Probability of Censoring Weights (IPCWs)

1) **Estimating the probability of being observed, in contrast to loss to follow-up** (given that the individual survived to the current wave and completed the assessment in the prior wave)

$$\Pr\,(observed)ij = \, logit(\Pr[A_{ij} = 0 \mid A_{i(j-1)} = 0,\, B_{i(j-1)}, C_{ij0},\, D_{i(j-1)}]) \qquad \text{Eq. (1)}$$

Where:

- A_{ij} equals to 0 if the individual is being observed at time *j*, and equals to 1 if the individual has censored due to loss to follow-up
- $B_{i(j-1)}$ represents memory scores at time *j-1*
- C_{ijo} represents job insecurity status at baseline
- $D_{i(j-1)}$ represents all covariates in the primary analytic models at time *j-1*

2) **Generating IPCWs:**

$$IPCW_{ij} = \frac{1}{\Pr\,(observed)_{ij}} \qquad \text{Eq. (2)}$$

Where:

- $IPCW_{ij}$ represents the inverse probability of censoring weight for individual *i* at time *j*.

© 2022 Yu X et al. *JAMA Network Open*.

eFigure. Study Design and Distribution of Study Sample From 2006-2016

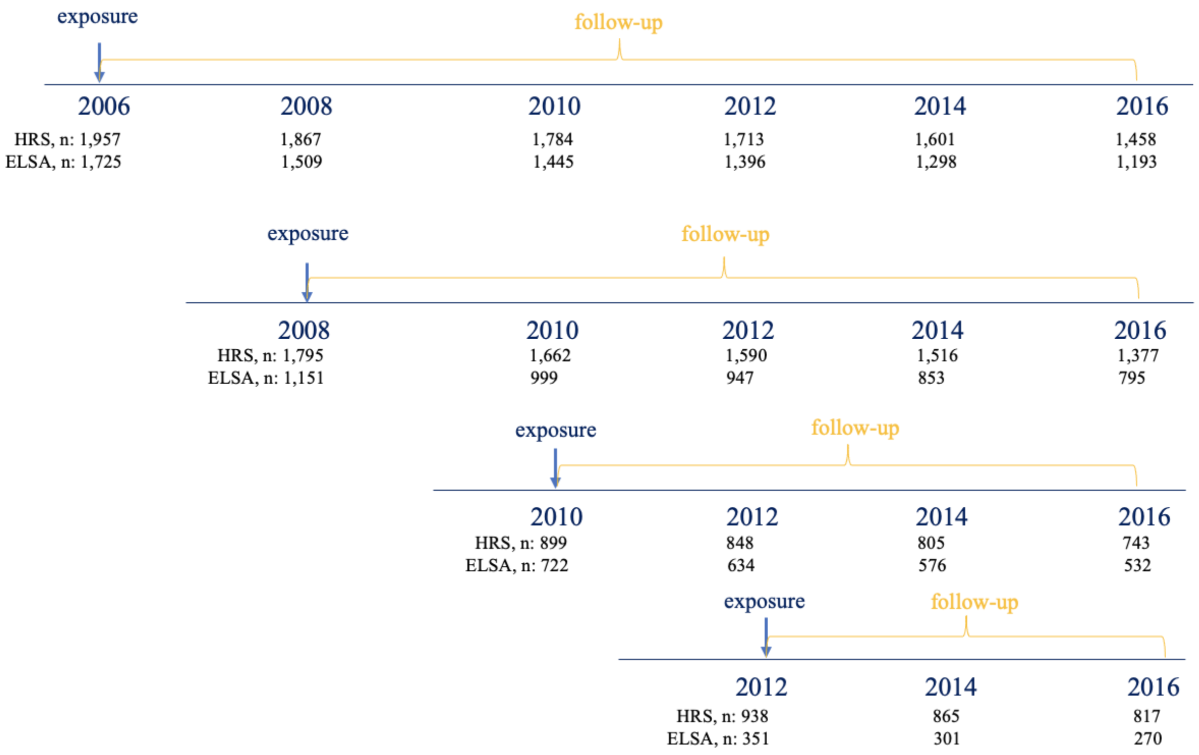

© 2022 Yu X et al. *JAMA Network Open*.

eTable 1. Classification and Definition of Occupations in HRS			
Occupation in HRS	ISCO-08 major group	Skill level	Occupation classification
01.management	1 manager	3+4	higher-skilled level
02.business operation specialists	2 professionals / 3 technicians and associate professionals	3 or 4	higher-skilled level
03.financial specialists	2 professionals / 3 technicians and associate professionals	3 or 4	higher-skilled level
04.computer and math	2 professionals / 3 technicians and associate professionals	3 or 4	higher-skilled level
05.architecture and engineering	2 professionals / 3 technicians and associate professionals	3 or 4	higher-skilled level
06.life/physical/social science	2 professionals / 3 technicians and associate professionals	3 or 4	higher-skilled level
07.community and social service	2 professionals / 3 technicians and associate professionals	3 or 4	higher-skilled level
08.legal	2 professionals / 3 technicians and associate professionals	3 or 4	higher-skilled level
09.education/training/library	2 professionals / 3 technicians and associate professionals	3 or 4	higher-skilled level
10.arts/design/entertainment	2 professionals / 3 technicians and associate professionals	3 or 4	higher-skilled level
11.healthcare practitioner/technician	2 professionals / 3 technicians and associate professionals	3 or 4	higher-skilled level
12.healthcare support	2 professionals / 3 technicians and associate professionals	3 or 4	higher-skilled level
13.protective service o	4 Clerical, sales and services occupation	2	lower-skilled level
14.food preparation and serving	4 Clerical, sales and services occupation	2	lower-skilled level
15.blding/grounds/clean/maintain	6 Skilled agricultural, forestry and fishery workers	2	lower-skilled level
16.personal care and service	4 Clerical, sales and services occupation	2	lower-skilled level
17.sales	5 services and sales workers	2	lower-skilled level
18.office and administrative support	4 Clerical, sales and services occupation	2	lower-skilled level
19.farm/fish/forestry occupations	6 Skilled agricultural, forestry and fishery workers	2	lower-skilled level
20.construction trades	7 Craft and related trades workers	2	lower-skilled level
21.extraction workers	7 Craft and related trades workers	2	lower-skilled level
22.install/maintain/repair workers	8 plant and machine operators, and assemblers	2	lower-skilled level
23.production	7 Craft and related trades workers	2	lower-skilled level
24.transport/material moving	8 plant and machine operators, and assemblers	2	lower-skilled level
25.military specific	0 armed forces occupation	1+2+4	lower-skilled level

International Standard Classification of Occupation (ISCO) has defined 10 major occupation groups (ISCO-08 major groups), which are classified into four skill levels (1, 2, 3, and 4). According to ISCO, we classified the HRS fielded 25 occupations into ISCO-08 major occupation groups, and defined them as higher-skilled level occupation (skill levels 3 and 4) and lower-skilled level occupation (skill levels 1 and 2)

© 2022 Yu X et al. *JAMA Network Open*.

eTable 2. Classification and Definition of Occupations in ELSA	
Occupation in ELSA	Skill level
1.employers in large organizations	higher-skilled level
2.higher managerial	higher-skilled level
3.higher professional	higher-skilled level
4.lower professional & higher technical	higher-skilled level
5.lower managerial occupations	higher-skilled level
6.higher supervisory occupations	higher-skilled level
7.intermediate	lower-skilled level
8.employers in small organizations	lower-skilled level
9.own account workers	lower-skilled level
10.lower supervisory	lower-skilled level
11.lower technical	lower-skilled level
12.semi-routine	lower-skilled level
13.routine	lower-skilled level

© 2022 Yu X et al. *JAMA Network Open*.

eTable 3. Sampling-Weighted Linear Mixed-Effects Regression Analyses of the Association Between Job Insecurity and Memory and Decline From 2006-2016, US (HRS) and England (ELSA) (N = 9538)

	Model 1				Model 2				Model 3			
	β (95% CI)			*P*	β (95% CI)			*P*	β (95% CI)			*P*
**Pooled analyses** with interaction between job insecurity and years of follow-up												
Job insecurity (yes vs. no)	-0.12	(-0.20 to	-0.04)	<0.001	-0.06	(-0.13 to	0.02)	0.12	-0.04	(-0.11 to	0.03)	0.30
Years of follow-up	-0.03	(-0.04 to	-0.02)	<0.001	-0.03	(-0.04 to	-0.02)	<0.001	-0.03	(-0.04 to	-0.02)	<0.001
Job insecurity ✕ Years of follow-up	0.01	(-0.01 to	0.01)	0.32	0.01	(-0.01 to	0.02)	0.28	0.01	(-0.01 to	0.01)	0.29
Years of follow-up^2	0.01	(-0.01 to	0.01)	0.82	0.01	(-0.01 to	0.01)	0.84	0.01	(-0.01 to	0.01)	0.83
**Pooled analyses** with interaction between job insecurity and country of residence												
Job insecurity (yes vs. no)	-0.02	(-0.08 to	0.04)	0.47	0.01	(-0.05 to	0.06)	0.89	0.02	(-0.04 to	0.08)	0.52
US (vs. England)	-0.09	(-0.13 to	-0.04)	<0.001	-0.28	(-0.32 to	-0.23)	<0.001	-0.25	(-0.30 to	-0.20)	<0.001
Job insecurity ✕ US	-0.08	(-0.18 to	0.02)	0.12	-0.04	(-0.13 to	0.05)	0.34	-0.03	(-0.12 to	0.06)	0.50
Years of follow-up	-0.03	(-0.04 to	-0.02)	<0.001	-0.03	(-0.04 to	-0.02)	<0.001	-0.03	(-0.04 to	-0.02)	<0.001
Years of follow-up^2	0.01	(-0.01 to	0.01)	0.84	0.01	(-0.01 to	0.01)	0.86	0.01	(-0.01 to	0.01)	0.85

Note: Memory scores were z-standardized with mean values and standard deviation at baseline.
Cluster and stratification were included to account for complex survey design in HRS and ELSA.
Model 1 adjusted for baseline year, baseline age, sex, marital status, race, and foreign-born status
Model 2 adjusted for baseline year, baseline age, sex, marital status, race, foreign-born status, occupation, wealth, and education.
Model 3 adjusted for baseline year, baseline age, sex, marital status, race, foreign-born status, occupation, wealth, education, alcohol consumption, smoking history, BMI, hypertension, diabetes, stroke, cardiovascular diseases, cancer, and depressive symptoms.

© 2022 Yu X et al. *JAMA Network Open*.

eTable 4. Attrition-Weighted Linear Mixed-Effects Regression Analyses of the Association Between Job Insecurity and Memory and Decline From 2006-2016, the US (HRS) and England (ELSA) (N = 9538)

	Model 1				Model 2				Model 3			
	β (95% CI)			*P*	β (95% CI)			*P*	β (95% CI)			*P*
**Pooled analyses** with interaction between job insecurity and years of follow-up												
Job insecurity (yes vs. no)	-0.11	(-0.15 to	-0.07)	<0.001	-0.06	(-0.10 to	-0.02)	<0.001	-0.04	(-0.08 to	-0.01)	0.04
Years of follow-up	-0.01	(-0.02 to	-0.01)	0.02	-0.01	(-0.02 to	-0.01)	0.01	-0.01	(-0.02 to	-0.01)	0.01
Job insecurity ✕ Years of follow-up	0.01	(-0.01 to	0.01)	0.11	0.01	(-0.01 to	0.01)	0.12	0.01	(-0.01 to	0.01)	0.12
Years of follow-up^2	-0.01	(-0.01 to	-0.01)	<0.001	-0.01	(-0.01 to	-0.01)	<0.001	-0.01	(-0.01 to	-0.01)	<0.001
**Pooled analyses** with interaction between job insecurity and country of residence												
Job insecurity (yes vs. no)	-0.04	(-0.10 to	0.02)	0.18	-0.01	(-0.07 to	0.04)	0.65	-0.01	(-0.05 to	0.05)	0.97
US (vs. England)	-0.12	(-0.15 to	-0.08)	<0.001	-0.24	(-0.28 to	-0.20)	<0.001	-0.21	(-0.25 to	-0.17)	<0.001
Job insecurity ✕ US	-0.10	(-0.17 to	-0.02)	0.01	-0.05	(-0.12 to	0.02)	0.16	-0.04	(-0.11 to	0.03)	0.26
Years of follow-up	-0.01	(-0.02 to	-0.01)	0.04	-0.01	(-0.02 to	-0.01)	0.02	-0.01	(-0.02 to	-0.01)	0.02
Years of follow-up^2	-0.01	(-0.01 to	-0.01)	<0.001	-0.01	(-0.01 to	-0.01)	<0.001	-0.01	(-0.01 to	-0.01)	<0.001

Note: Memory scores were z-standardized according to the mean and standard deviation at baseline.
A total of 568 observations from individuals lost to follow-up at prior time points were not included because of missing IPCWs.
IPCWs weights were included to account for potential selective attrition.
Model 1 adjusted for baseline year, baseline age, sex, marital status, race, and foreign-born status
Model 2 adjusted for baseline year, baseline age, sex, marital status, race, foreign-born status, occupation, wealth, and education.
Model 3 adjusted for baseline year, baseline age, sex, marital status, race, foreign-born status, occupation, wealth, education, alcohol consumption, smoking history, BMI, hypertension, diabetes, stroke, cardiovascular diseases, cancer, and depressive symptoms.

© 2022 Yu X et al. *JAMA Network Open*.

eTable 5. Restricted Multivariable-Adjusted Linear Mixed-Effects Regression Analyses of the Association Between Job Insecurity and Memory and Decline Among Individuals Aged 55-64 From 2006-2016, the US (HRS) and England (ELSA) (N = 7295)												
	Model 1				Model 2				Model 3			
	β (95% CI)			*P*	β (95% CI)			*P*	β (95% CI)			*P*
**Pooled analyses** with interaction between job insecurity and years of follow-up												
Job insecurity (yes vs. no)	-0.09	(-0.13 to	-0.04)	<0.001	-0.04	(-0.09 to	0.01)	0.06	-0.03	(-0.07 to	0.02)	0.22
Years of follow-up	-0.01	(-0.01 to	0.01)	0.89	-0.01	(-0.01 to	0.01)	0.82	-0.01	(-0.01 to	0.01)	0.78
Job insecurity ✕ Years of follow-up	0.01	(-0.01 to	0.01)	0.71	0.01	(-0.01 to	0.010	0.73	0.01	(-0.01 to	0.01)	0.74
Years of follow-up^2	-0.01	(-0.01 to	-0.01)	0.00	-0.01	(-0.01 to	-0.01)	0.00	-0.01	(-0.01 to	-0.01)	<0.001
**Pooled analyses** with interaction between job insecurity and country of residence												
Job insecurity (yes vs. no)	-0.04	(-0.10 to	0.02)	0.19	-0.01	(-0.06 to	0.040	0.71	0.01	(-0.05 to	0.05)	0.99
US (vs. England)	-0.13	(-0.18 to	-0.09)	<0.001	-0.26	(-0.30 to	-0.22)	<0.001	-0.23	(-0.28 to	-0.19)	<0.001
Job insecurity ✕ US	-0.08	(-0.17 to	-0.01)	0.04	-0.06	(-0.13 to	0.02)	0.15	-0.05	(-0.12 to	0.03)	0.21
Years of follow-up	-0.01	(-0.01 to	0.01)	0.95	-0.01	(-0.01 to	0.01)	0.88	-0.01	(-0.01 to	0.01)	0.84
Years of follow-up^2	-0.01	(-0.01 to	-0.01)	<0.001	-0.01	(-0.01 to	-0.01)	0.00	-0.01	(-0.01 to	-0.01)	<0.001

Note: Memory scores were z-standardized according to the mean and standard deviation at baseline.
Model 1 adjusted for baseline year, baseline age, sex, marital status, race, and foreign-born status
Model 2 adjusted for baseline year, baseline age, sex, marital status, race, foreign-born status, occupation, wealth, and education.
Model 3 adjusted for baseline year, baseline age, sex, marital status, race, foreign-born status, occupation, wealth, education, alcohol consumption, smoking history, BMI, hypertension, diabetes, stroke, cardiovascular diseases, cancer, and depressive symptoms.

© 2022 Yu X et al. *JAMA Network Open*.

eTable 6. Restricted Multivariable-Adjusted Linear Mixed-Effects Regression Analyses of the Association Between Job Insecurity and Memory and Decline Among Participants With at Least Upper Secondary and Vocational Training From 2006-2016, the US (HRS) and England (ELSA) (N = 7449)

	Model 1				Model 2				Model 3			
	β (95% CI)			*P*	β (95% CI)			*P*	β (95% CI)			*P*
**Pooled analyses** with interaction between job insecurity and years of follow-up												
Job insecurity (yes vs. no)	-0.07	(-0.12 to	-0.03)	<0.001	-0.04	(-0.08 to	0.01)	0.11	-0.02	(-0.07 to	0.03)	0.38
Years of follow-up	-0.01	(-0.02 to	-0.01)	<0.001	-0.01	(-0.02 to	-0.01)	<0.001	-0.01	(-0.02 to	-0.01)	<0.001
Job insecurity ✕ Years of follow-up	0.01	(-0.01 to	0.01)	0.36	0.01	(-0.01 to	0.01)	0.36	0.01	(-0.01 to	0.01)	0.35
Years of follow-up^2	-0.01	(-0.01 to	-0.01)	<0.001	-0.01	(-0.01 to	-0.01)	<0.001	-0.01	(-0.01 to	-0.01)	<0.001
**Pooled analyses** with interaction between job insecurity and country of residence												
Job insecurity (yes vs. no)	-0.02	(-0.08 to	0.05)	0.60	0.01	(-0.06 to	0.07)	0.81	0.02	(-0.04 to	0.08)	0.55
US (vs. England)	-0.21	(-0.26 to	-0.17)	<0.001	-0.24	(-0.28 to	-0.20)	<0.001	-0.21	(-0.25 to	-0.16)	<0.001
Job insecurity ✕ US	-0.07	(-0.15 to	0.01)	0.11	-0.05	(-0.13 to	0.03)	0.19	-0.05	(-0.13 to	0.03)	0.26
Years of follow-up	-0.01	(-0.02 to	-0.01)	<0.001	-0.01	(-0.02 to	-0.01)	<0.001	-0.01	(-0.02 to	-0.01)	<0.001
Years of follow-up^2	-0.01	(-0.01 to	-0.01)	<0.001	-0.01	(-0.01 to	-0.01)	<0.001	-0.01	(-0.01 to	-0.01)	<0.001

Note: Memory scores were z-standardized according to the mean and standard deviation at baseline.
Model 1 adjusted for baseline year, baseline age, sex, marital status, race, and foreign-born status
Model 2 adjusted for baseline year, baseline age, sex, marital status, race, foreign-born status, occupation, wealth, and education.
Model 3 adjusted for baseline year, baseline age, sex, marital status, race, foreign-born status, occupation, wealth, education, alcohol consumption, smoking history, BMI, hypertension, diabetes, stroke, cardiovascular diseases, cancer, and depressive symptoms.

© 2022 Yu X et al. *JAMA Network Open*.

eTable 7. Restricted Multivariable-Adjusted Linear Mixed-Effects Regression Analyses of the Association Between Job Insecurity and Memory and Decline Among Participants With No History of Diabetes, Hypertension, Stroke, Cardiovascular Disease, Cancer, and Depressive Symptoms at Baseline, the US (HRS) and England (ELSA) (N = 3990)

	Model 1				Model 2				Model 3			
	β (95% CI)			*P*	β (95% CI)			*P*	β (95% CI)			*P*
**Pooled analyses** with interaction between job insecurity and years of follow-up												
Job insecurity (yes vs. no)	-0.09	(-0.16 to	-0.03)	0.01	-0.05	(-0.12 to	0.01)	0.11	-0.05	(-0.11 to	0.01)	0.13
Years of follow-up	-0.01	(-0.02 to	0.01)	0.16	-0.01	(-0.02 to	0.01)	0.12	-0.01	(-0.02 to	0.01)	0.12
Job insecurity × Years of follow-up	0.01	(-0.01 to	0.01)	1.00	0.01	(-0.01 to	0.01)	0.96	0.01	(-0.01 to	0.01)	0.97
Years of follow-up^2	-0.01	(-0.01 to	0.01)	0.08	-0.01	(-0.01 to	0.01)	0.09	-0.01	(-0.01 to	0.01)	0.09
**Pooled analyses** with interaction between job insecurity and country of residence												
Job insecurity (yes vs. no)	-0.06	(-0.14 to	0.02)	0.14	-0.03	(-0.11 to	0.04)	0.41	-0.03	(-0.10 to	0.04)	0.42
US (vs. England)	-0.06	(-0.12 to	-0.01)	0.03	-0.21	(-0.27 to	-0.15)	<0.001	-0.20	(-0.26 to	-0.14)	<0.001
Job insecurity × US	-0.08	(-0.20 to	0.04)	0.20	-0.04	(-0.16 to	0.07)	0.43	-0.04	(-0.15 to	0.07)	0.48
Years of follow-up	-0.01	(-0.02 to	0.01)	0.15	-0.01	(-0.02 to	0.01)	0.12	-0.01	(-0.02 to	0.01)	0.11
Years of follow-up^2	-0.01	(-0.01 to	0.01)	0.08	-0.01	(-0.01 to	0.01)	0.09	-0.01	(-0.01 to	0.01)	0.10

Note: Memory scores were z-standardized according to the mean and standard deviation at baseline.
Model 1 adjusted for baseline year, baseline age, sex, marital status, race, and foreign-born status.
Model 2 adjusted for baseline year, baseline age, sex, marital status, race, foreign-born status, occupation, wealth, and education.
Model 3 adjusted for baseline year, baseline age, sex, marital status, race, foreign-born status, occupation, wealth, education, alcohol consumption, smoking history, and BMI.

© 2022 Yu X et al. *JAMA Network Open*.

eTable 8. Restricted Multivariable-Adjusted Linear Mixed-Effects Regression Analyses of the Association Between Job Insecurity and Memory and Decline Among Participants That Were Not Dismissed Before Baseline, 2006-2016, the US (HRS) and England (ELSA) (N = 8232)

	Model 1				Model 2				Model 3			
	β (95% CI)			*P*	β (95% CI)			*P*	β (95% CI)			*P*
**Pooled analyses** with interaction between job insecurity and years of follow-up												
Job insecurity (yes vs. no)	-0.12	(-0.16 to	-0.07)	<0.001	-0.06	(-0.11 to	-0.02)	0.01	-0.05	(-0.09 to	-0.01)	0.04
Years of follow-up	-0.01	(-0.02 to	0.01)	0.07	-0.01	(-0.02 to	.0.01)	0.06	-0.01	(-0.02 to	-0.01)	0.05
Job insecurity ✕ Years of follow-up	0.01	(0.01 to	0.01)	0.04	0.01	(0.01 to	0.01)	0.04	0.01	(0.01 to	0.01)	0.04
Years of follow-up^2	-0.01	(-0.01 to	-0.01)	<0.001	-0.01	(-0.01 to	-0.01)	<0.001	-0.01	(-0.01 to	-0.01)	<0.001
**Pooled analyses** with interaction between job insecurity and country of residence												
Job insecurity (yes vs. no)	-0.05	(-0.10 to	0.01)	0.09	-0.02	(-0.07 to	0.03)	0.42	-0.01	(-0.06 to	0.04)	0.65
US (vs. England)	-0.12	(-0.16 to	-0.07)	<0.001	-0.24	(-0.29 to	-0.20)	0.00	-0.22	(-0.26 to	-0.17)	<0.001
Job insecurity ✕ US	-0.10	(-0.18 to	-0.02)	0.02	-0.04	(-0.11 to	0.04)	0.32	-0.03	(-0.10 to	0.05)	0.50
Years of follow-up	-0.01	(-0.01 to	0.01)	0.16	-0.01	(-0.01 to	0.01)	0.12	-0.01	(-0.01 to	0.01)	0.11
Years of follow-up^2	-0.01	(-0.01 to	-0.01)	<0.001	-0.01	(-0.01 to	-0.01)	<0.001	-0.01	(-0.01 to	-0.01)	<0.001

Note: Memory scores were z-standardized according to the mean and standard deviation at baseline.
Model 1 adjusted for baseline year, baseline age, sex, marital status, race, and foreign-born status
Model 2 adjusted for baseline year, baseline age, sex, marital status, race, foreign-born status, occupation, wealth, and education.
Model 3 adjusted for baseline year, baseline age, sex, marital status, race, foreign-born status, occupation, wealth, education, alcohol consumption, smoking history, BMI, hypertension, diabetes, stroke, cardiovascular diseases, cancer, and depressive symptoms.

© 2022 Yu X et al. *JAMA Network Open*.

eTable 9. Baseline Characteristics of the Included Study Sample and Excluded Individuals, the US (HRS) and England (ELSA), 2006-2016

Characteristics	Included (N=9,538)	Excluded (N=29312)	P-value
Memory scores, mean (SD)	11.19 (3.06)	9.51 (3.57)	<0.01[a]
Age, mean (SD)	60.97 (6.06)	66.33(12.13)	<0.01[a]
Age, median	59	66	
Age, range	55-97	18-105	
Female (vs. Male), n (%)	4,981 (52.22)	17,096 (58.32)	0.02[b]
White (vs. Others [d]), n (%)	8,196 (85.93)	23,157 (79.01)	<0.01[b]
Foreign-born status, n (%)	383 (4.02)	832(2.84)	<0.01[b]
Marital status, n (%)			
Partnered	7,157 (75.04)	18,708 (63.84)	<0.01[b]
Separated/divorce	1,316 (13.80)	3,606 (12.30)	
Widowed	636 (6.67)	5,525 (18.85)	
Never married	429 (4.50)	1,467 (5.01)	
Education, n (%)			
Less than upper secondary	1,345 (14.10)	8,218 (28.04)	<0.01[c]
Upper secondary and vocational training	5,007 (52.50)	14,631 (49.92)	
Tertiary	2,442 (25.60)	4,550 (15.52)	
Household wealth (in quintile), n (%)			
1st (the poorest)	1,596 (16.73)	6,918 (23.67)	<0.01[c]
2nd	1,798 (18.85)	6,169 (21.11)	
3rd	2,022 (21.20)	5,498 (18.82)	
4th	2,045 (21.44)	5,370 (18.38)	
5th (the richest)	2,031 (21.29)	5,266 (18.02)	
Occupation (Higher-skilled vs. lower-skilled), n (%)	3,631 (38.07)	4,893 (32.47)	<0.01[b]
Smoking history (Ever vs. never), n (%)	5,375 (56.35)	17,161 (58.99)	0.02[b]
Alcohol assumption (yes vs. no), n (%)	7,159 (75.06)	16,602 (56.64)	0.64[b]
BMI, n (%)			
<18.5	70 (0.73)	444 (1.53)	<0.01[c]
18.5-24.9	2,269 (23.79)	7,563 (26.12)	
25.0-29.9	3,576 (37.49)	10,131 (34.99)	
>29.9	3,599 (37.73)	10,812 (37.35)	
Hypertension (yes vs. no), n (%)	3,859 (40.46)	15,050 (51.36)	0.07[b]
Diabetes (yes vs. no), n (%)	1,087 (11.40)	5,094 (17.38)	<0.01[b]
stroke (yes vs. no), n (%)	230 (2.41)	2,309 (7.88)	0.35[b]
Cardiovascular disease (yes vs. no), n (%)	1,139 (11.94)	6,328 (21.60)	0.04[b]
Cancer (yes vs. no), n (%)	734 (7.70)	3,378 (11.53)	0.62[b]
Depressive symptoms (yes vs. no), n (%)	1,355 (14.21)	5,113 (20.30)	<0.01[b]

Note: [a]t test. [b] Pearson chi-square test. [c] Wilcoxon rank-sum tests. [d] Other racial/ethnic groups in the HRS included Black/African American, American Indian or Alaskan Native, Asian or Pacific Islander, Hispanic/Latino, combination of Black and American Indian, and other; other racial/ethnic groups in the ELSA included Mixed ethnic group, Black, Black British, Asian, Asian British, and any other group.

© 2022 Yu X et al. *JAMA Network Open*.
